# Can sexual health interventions make community-based health systems more responsive to adolescents? A realist informed study in rural Zambia

**DOI:** 10.1186/s12978-019-0847-x

**Published:** 2020-01-08

**Authors:** Chama Mulubwa, Anna-Karin Hurtig, Joseph Mumba Zulu, Charles Michelo, Ingvild Fossgard Sandøy, Isabel Goicolea

**Affiliations:** 1 School of Public Health, University of Zambia, Lusaka, Zambia; 2 Zambart Project, University of Zambia, Lusaka, Zambia; 3 Department of Epidemiology and Global Health, Umeå University, SE Umeå, Sweden; 4 Centre for Intervention Science in Maternal and Child Health (CISMAC), Centre for International Health (CIH), Department of Global Public Health and Primary Care, University of Bergen, Bergen, Norway

**Keywords:** Adolescents, Community-based health system, Intervention−context−actors−mechanism−outcomes, Sexual reproductive health and rights, Realist evaluation, Retroduction

## Abstract

**Introduction:**

Community-based sexual reproductive interventions are key in attaining universal health coverage for all by 2030, yet adolescents in many countries still lack health services that are responsive to their sexual reproductive health and rights’ needs. As the first step of realist evaluation, this study provides a programme theory that explains how, why and under what circumstances community-based sexual reproductive health interventions can transform (or not) ‘ordinary’ community-based health systems (CBHSs) into systems that are responsive to the sexual reproductive health of adolescents.

**Methods:**

This realist approach adopted a case study design. We nested the study in the full intervention arm of the Research Initiative to Support the Empowerment of Girls trial in Zambia. Sixteen in-depth interviews were conducted with stakeholders involved in the development and/or implementation of the trial. All the interviews were recorded and analysed using NVIVO version 12.0. Thematic analysis was used guided by realist evaluation concepts. The findings were later synthesized using the Intervention−Context−Actors−Mechanism−Outcomes conceptualization tool. Using the retroduction approach, we summarized the findings into two programme theories.

**Results:**

We identified two initial testable programme theories. The first theory presumes that adolescent sexual reproductive health and rights (SRHR) interventions that are supported by contextual factors, such as existing policies and guidelines related to SRHR, socio-cultural norms and CBHS structures are more likely to trigger mechanisms among the different actors that can encourage uptake of the interventions, and thus contribute to making the CBHS responsive to the SRHR needs of adolescents. The second and alternative theory suggests that SRHR interventions, if not supported by contextual factors, are less likely to transform the CBHSs in which they are implemented. At individual level the mechanisms, awareness and knowledge were expected to lead to value clarification’, which was also expected would lead to individuals developing a ‘supportive attitude towards adolescent SRHR. It was anticipated that these individual mechanisms would in turn trigger the collective mechanisms, communication, cohesion, social connection and linkages.

**Conclusion:**

The two alternative programme theories describe how, why and under what circumstances SRHR interventions that target adolescents can transform ‘ordinary’ community-based health systems into systems that are responsive to adolescents.

## Plain English summary

In Zambia, just as in other low- and middle-income countries, a good number of adolescents lack access to health services that consider their sexual reproductive health and rights (SRHR) needs with respect. In low- and middle-income countries, a community-based health system (CBHS) approach is promising in making reproductive health-related services friendlier toward adolescents. In our study, we defined the CBHS as all individuals, places, relationships and processes involved in ‘speaking for’ and supporting SRHR of adolescents in the communities, and are at the same time linked to the local health facility. We nested this study in an ongoing project that was researching the initiative to support and empower girls in Zambia. The aim of our study was to develop a set of assumptions that explain how sexual reproductive health interventions can make the CBHS in which they are conducted more friendly to adolescents. In our results, we came up with two sets of assumptions, which we plan to test in the future. The first set of assumptions suggests that interventions targeting adolescents’ SRHR can only work if they are supported by conditions such as rules and guidelines that exist in the communities in which they are implemented. Some of the things that can make such interventions work (or not) include individuals developing awareness and knowledge, developing a supportive attitude and communication. The assumptions reported in this study suggest how, why and under what conditions sexual reproductive health-related interventions that target adolescents can work or not.

## Background

In 2015, 226 million young people aged 15–24 lived in Africa, accounting for 19% of the global youth population [1]. Data from sub-Saharan Africa suggest that, while a significant number of adolescents have their first sexual experience at an early age (ranging from 2.0 to 27.0% of adolescents under age 15) [2], in many cases they do not use any form of protection to prevent pregnancy or sexually transmitted infections [3, 4]. In addition, not all sexual experiences among adolescents are consensual, since the prevalence of coerced sex and sexual abuse at this age remains high (approximately 16% in Zambia) [5, 6].

Adolescents’ SRHR needs differ from those of adults, and their access to services is poorer [5]. In low- and middle-income countries, adolescents’ access to SRHR services is further hindered by issues such as poverty, feeble health systems, gender-based violence, abuse, forced marriages and cultural norms [7, 8]. To address these challenges, the World Health Organization (WHO) calls for ensuring that health-care services are youth-friendly; that is, they are available, accessible, acceptable and equitable for diverse youth sub-populations [9]. Despite the relevance of this approach for challenging adult-centred health care, a number of limitations have been pointed out. First, youth-friendly sexual reproductive health services have not worked as anticipated. Availability, access and utilization of these services in low- and middle-income countries have been criticized for not being comprehensive. Other factors affecting utilization include health workers refusing to give unmarried adolescents contraceptive information due to socio-cultural norms, contraceptives out of stock, long distances to the health facility, and unfriendly service provision [10, 11]. Second, if youth-friendly services exist at all, they have been limited to the health-care facilities in many settings, disregarding that youth sexual and reproductive health needs might demand the involvement of other sectors and arenas, i.e. schools and communities [12, 13].

In order to address this gap, WHO (in 2015) recommended a transition from adolescent-friendly projects to adolescent-responsive health systems [14]. WHO defined an adolescent-responsive health system as a system that goes beyond sexual reproductive health to address the full range of adolescents’ development needs (including information giving and building skills), using diverse platforms such as public and private facilities, schools, youth centres and outreach strategies [14, 15]. This includes and is especially relevant in low- and middle-income countries, working with communities in strengthening the CBHSs and making them responsive to the SRHR of adolescents.

In this study, we follow Schneider and Lehmann’s definition of a CBHS as ‘a set of local actors, relationships, and processes engaged in producing, advocating for, and supporting health in communities and households outside of, but existing in relationship to, formal health structures' (pg. 114) [16]. This definition also includes the engagement of all the actors relevant to address the public health issues at hand, and considers the local values and culture [16]. The concept of using a CBHS approach to address SRHR issues is not new, especially in low- and middle-income countries, but the urgency in taking the systems approach for community health has now been recognized as a key feature in attaining universal health coverage by 2030 [17]. A systems approach for community health recognizes that CBHSs are made up of components that are interactive in nature and interdependent on both external and internal factors that exist in a CBHS. In the last decade, the CBHS approach has been key in delivering services related to SRHR, such as HIV and AIDS, maternal and child health and contraceptive use [18, 19]. Among key actors that engage in delivering CBHS services included community-based health workers (CBHWs), caregivers, health providers, existing government and non-governmental organizations, and representatives from local health and political structures [16].

A few studies examining programmes targeting the components of a CBHS have demonstrated positive outcomes. For example: evidence from a study conducted in Ethiopia among respondents of reproductive age (including adolescents) show that CBHWs can play a significant role in expanding contraceptive use in low- and middle-income countries [20]. Evidence from Ghana suggests that adolescent SRHR activities should also target parents (and community members) as a way of breaking socio-cultural barriers [21]. School programmes have been shown to be more effective in empowering adolescents in relation to their SRHR when linked to the community [22, 23]. It is evident from the mentioned studies that addressing adolescent SRHR can only be achieved through multi-sectorial approaches that involve working with all relevant stakeholders, including parents, community members and policymakers; in other words, the CBHS [23, 24]. However, there is still a critical gap in our knowledge of how interventions that target the CBHS can contribute to making it responsive towards the SRHR of adolescents.

### The community-based health system in Zambia

The general definition of CBHS tends to be similar worldwide. The characteristics that define the CBHSs include the communities in which they exist, the historic, economic and political systems, and the social and cultural norms existing in these communities [16]. Community-based SRHR services in Zambia are mainly offered through the CBHWs. Such SRHR services are primarily focused on family planning and HIV and mainly target adults (married) or those with children. In Zambia, each CBHS consists of the health post or health centre serving approximately 500–1000 households. The health centres’ and health posts work in partnership with the local community mainly through CBHWs. It is in this setting of the CBHS that the interventions of the project called ‘the Research Initiative to Support the Empowerment of Girls (RISE)’ was implemented.

### The RISE intervention: an opportunity to make CBHSs more responsive towards adolescents

The main aim of the RISE project was to test interventions for enhancing opportunities for communities to support adolescent girls to continue going to school, and for increasing girls’ possibilities to postpone pregnancy and marriage. Details of the RISE project have been reported elsewhere [25, 26]. Briefly, RISE is a three-arm cluster randomized controlled trial involving 157 schools and targeting approximately 4900 girls. In 2016, the trial enrolled girls who were in grade 7 (average age approximately 14 years). The interventions were provided for two years from Septemebr, 2016 to Novermber, 2018 [25, 26].

Our study focused on the full intervention arm of RISE referred to as the ‘combined intervention arm’. The combined intervention arm used schools and communities as the arenas for implementation. The intervention brought together CBHWs and teachers to coordinate: 1) community and parental meetings promoting supportive social norms around the postponement of early marriage and early childbearing, as well as promoting education for girls. The community meetings were held twice per term in each school; 2) the establishment of new adolescents’ clubs in order to increase life skills and knowledge of SRHR, including modern contraceptives and promoting a change in behaviour and beliefs relating to contraceptive use among both school-going and non-school-going adolescent girls and boys; 3) providing limited school material support (books and pens); and 4) providing economic support of paying school fees, limited monthly financial support to girls, and annual financial support to families [26]. Through these activities and a multi-sectorial approach of engaging all the stakeholders (adolescents, parents, teachers, CBHWs), the combined intervention arm of RISE aimed to improve girls’ SRHR and decrease unwanted pregnancies. The RISE trial also had other arms that are not part of this study: the control arm, which only provided limited school material support; and the economic arm, which provided material and economic support.

In order to improve girls’ SRHR, interventions such as RISE have to change the approach of the CBHS into a system that is responsive towards adolescents’ SRHR needs. Since “transformation of the CBHS” is an implicit outcome of complex interventions such as RISE, we considered the combined intervention arm as a good example of an SRHR intervention targeting all key arenas in the CBHS (school, community and health facility) where adolescents work, study, play and live. Consequently, the aim of this study is to understand how, why and under what circumstances an intervention that focuses on implementing youth clubs and community dialogue meetings coordinated by trained teachers and CBHWs is expected to transform an ‘ordinary’ CBHS into a system that is responsive to the SRHR of adolescents in rural Zambia. To answer the aim, we developed two initial programme theories. Programme theories can be used as a planning tool or as an evaluation tool [27]. The program theory developed in this article is the first step in an ongoing evaluation and will be tested in subsequent steps. Realist evaluation relies on eliciting program theories to understand interventions, so we consider that the programme theory presented in this study can be a useful tool for planners, implementers and evaluators of similar adolescents SRHR interventions, especially in the context of Sub Saharan Africa.

### Methodological approach

#### Realist evaluation

Realist evaluation is a theory-driven approach suitable to explore why, how and under what circumstances complex interventions such as the combined intervention arm of RISE succeed or fail [28, 29]. Realist evaluation recognizes that interventions and actors interact within their social reality, which also influences how the intervention is implemented and how the actors respond (or not) to the resources offered by the intervention [30]. Realist evaluation involves exploring and identifying the mechanisms by which the inputs are transformed into outputs and recognizes the need for specific conditions (or contexts) for this to occur [27, 30, 31]. Context is a key concept in realist evaluation, since different mechanisms can be triggered depending on different contextual factors. That makes it well suited for evaluating community-based interventions, which are most likely affected by the social and cultural norms in the community [28, 29].

The first step in realist evaluation is to develop the programme theory about how and why and for whom and under what circumstances a programme, or an intervention, works (or not). A programme theory is a ‘set of explicit and implicit assumptions or ideas’ of how the programme is expected to work. It provides the conceptual framework that links the intervention components to the outcomes and explains how and why the outcomes are expected to occur [27, 28]. In this programme theory, we recognize the role of actors who are also key but have not been so central in previous studies developing programme theories (for an important exception see Van Belle and Mukumbang et al., [27].

## Methods

### Study design

In this study we have used the combined intervention arm of the RISE as a case in order to develop a programme theory Developing the programme theory is essential to understanding how the key stakeholders expected the different elements of the intervention would work. Documenting and understanding the assumptions of the initial programme theory provides valuable lessons, not only for the interpretation of the outcomes of the RISE trial, but also to other stakeholders implementing adolescent sexual health programmes in Zambia and other low- and middle-income countries.

### Data collection

Data collection took place from April to September 2018. Sixteen interviews were conducted with key stakeholders. Participants were selected purposively as being the most relevant for this study due to their work on the RISE project and involvement in the development and/or implementation of RISE (Table 1). Nine of the 16 participants were men and seven were women. Ten participants were aged between 25 and 35, two were in their early 40s and one was in the early 50s.

**Table 1:** Summary characteristics of the stakeholders interviewed

Participants	Role on RISE
Researchers	Key stakeholders who had been actively involved in the initial implementation of the RISE but were no longer actively engaged in the implementation at the time of the interview. These were selected as they had played a key role in formulating the intervention, developing the proposal and the study tools.
Key stakeholders from the Ministry of Health	These stakeholders were working for the Ministry of Health and with adolescent SRH activities at national level.
Trial supervisors	The trial supervisors over saw the project intervention and activities at district level and were involved in the day to day implementation of the interventions. They worked closely with the country coordinator, the teachers and the CBHWs.
Principle investigators	Overseeing that all the activities related to RISE were conducted in line with the protocol. The principle investigators were deeply involved in the daily management of RISE.
Research team	These participants were part of the country research team focusing mainly on the implementation of the combined intervetion arm and on issues related to community engagement and school dropouts.

After providing informed consent, all the 16 individuals who were contacted agreed to participate. Of the contacted individuals, 11 were available to have the interview in person, four were only available by phone and one was only available through a Skype call. We conducted the interviews until no new information to answer our research question was being obtained [31]. As all the respondents were fluent in English, the first author conducted all the interviews in English using an open interview guide.

First, five interviews were conducted. The first five interviews were then transcribed and analysed. The results from the preliminary analysis were then used to inform data collection of the other set of interviews, following an emergent design [32]. The interview guide covered broad topics on the history of RISE and the RISE interventions but focused on how the combined intervention arm was being implemented, and how and why the intervention was expected to work to lead to the outcomes of this study. The interview guide also contained questions regarding the contexts in which the intervention was expected to work (or not). Towards the end of each interview, we used a ‘conceptual map’ to help the participants conceptualize how we had defined the CBHS. The conceptual map depicted all the intervention inputs, the actors involved in the implementation process and the anticipated outcomes of the project. The conceptual map helped the researcher to explain to the participants the intervention components in relation to the CBHS and to solicit for mechanisms.

### Data analysis

All the audio-recorded interviews were transcribed verbatim and exported to NIVIVO version 12.0 for handling the coding process. Thematic analysis was used [33], guided by realist evaluation of context−mechanisms−outcomes (CMOs) and later subdivided using the intervention−context−actors−mechanisms−outcomes (ICAMOs) conceptualization tool [30]. Data analysis included the following activities:

- The first author who conducted the interviews read through the transcripts to develop initial codes (inductively, line by line). The initial codes were then discussed with one of the co-authors and mapped using the realist framework of CMOs.
- Next, the codes were merged into subthemes and arranged according to their references to the context, mechanisms and outcomes.
- Through a group discussion with co-authors, we used the identified codes and themes to develop the first draft of the initial programme theory. During this discussion, we also subdivided the identified themes according to the different actors (adolescents, teachers, CBHWs, parents/guardians and community members) who participated in the interventions. The first draft of the initial programme theory was then presented during a workshop to a group of independent researchers who were not part of the study team but have had experience in using realist evaluation. This discussion helped the authors to agree on the themes that should be included.
- We then continued coding, merging the codes into subthemes and later into the major themes, which were used to refine the initial programme theory.
- We used a retroduction approach to develop the mechanisms that are theorized to lead to the outcomes within the specific actors. According to Easton [34], and applied by Mukumbang et al., [26, 30] ‘retroduction is a mode of analysis in which events are explained by identifying mechanisms which are capable of producing them’ [27]. In this study, we brought together codes that related to different mechanisms and different actors to develop the themes. We focused on bringing both the agreeing and contradictory codes under the same theme if they spoke to that specific theme, in order to best capture nuances. The identified mechanisms in this study were then used to explain how an ‘ordinary’ CBHS can be transformed to a system that is responsive to adolescent SRHR.

### Ethical approval

Ethical approval for this study was granted by the Excellence in Research Ethics and Science (ERES) committee in Lusaka, Zambia (approval number 2018-Jan-007). The selected key informants were informed that participation in the study was voluntary and would not affect their further work with RISE. Written informed consent and consent to audio-record the interviews was provided by all study participants. To ensure confidentiality, all personal identifiers were erased from the interview.

## Results

**Fig. 1:**
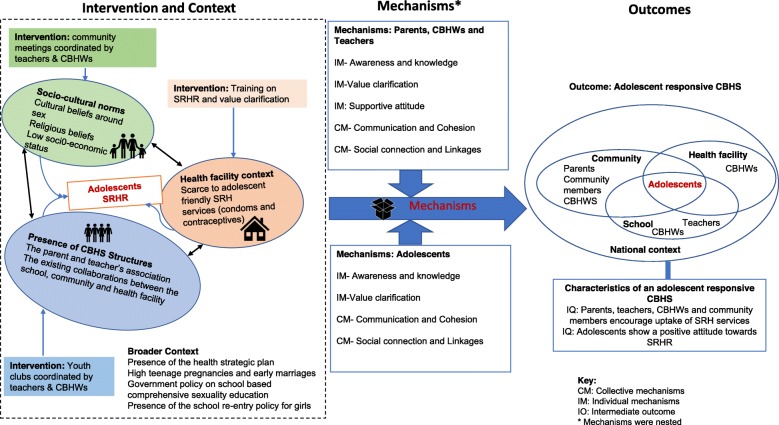
Schematic representation of the intervention−context−actors−mechanisms and outcomes

The results section, summarized in Fig. 1, will firstly describe the context, which is the conditions that the participants considered relevant to understand the transformation of the CBHS. We highlight the levels at which these contextual factors interact and how they can promote or hinder the transformation of a CBHS into a system that is responsive to adolescents. Secondly, we present the expected outcomes (as defined in this study), from the perspective of the participants. Lastly, we will describe the mechanisms, which include all factors expected to be operationalized within the actors participating in the various components of the intervention to produce the outcomes. While some mechanisms are expected to be triggered individually for the different actors (parents, youth, CBHWs, teachers and health-care workers), others are assumed to develop within interrelationships [9, 29]. In this study, the mechanisms anticipated to be triggered within interrelationships will be referred to as ‘collective mechanisms’.

## Context

The contextual conditions were grouped into four dimensions: presence of policies and guidelines related to SRHR; socio-cultural norms; presence of the CBHS structures; and health facility environment.

### Presence of policies and guidelines

Participants considered the presence of policies around the SRHR of adolescents as one contextual factor that provided both enabling and inhibiting conditions. Two policies were identified as key by the participants, the school re-entry policy and the presence of comprehensive sexuality education guidelines.

#### School re-entry policy

The school re-entry policy allows pregnant pupils in Zambia to continue attending school and also allows girls who drop-out of school to return back to schools after giving birth. The presence of this policy within the Ministry of General Education and school level meant messages in the interventions that promoted contraceptive use and family planning were expected to be acceptable as they would be very helpful to school-going girls who might have already given birth.

#### Presence of comprehensive sexuality education guidelines

Another government policy that the participants considered influential was the presence of the guidelines to teach comprehensive sexuality education in all schools in Zambia. Although participants highlighted that most schools had not started to implement comprehensive sexuality education, the presence of the guidelines in the schools meant that interventions that aimed to teach SRHR would probably be more easily accepted at school level. The guidelines on comprehensive sexuality education also provided a foundation for the development of the intervention manuals, thus providing an enabling environment in which CBHS interventions that aim to promote SRHR for adolescents could ‘ride on’, as one participant explained:

*“I will give an example of the Ministry of Education: we did look at their comprehensive sexuality education curriculum when we were developing our own manual and they were very helpful in guiding us on what to include … and also we did involve them [referring to the Ministry of General Education stakeholders] and they did a review so I feel we tried as much as possible to be within their scope”’ Key Informant Interview 6.*

Despite the presence of the guidelines, most of the participants also reported limited access to comprehensive SRHR information in most Zambian schools. This was worsened by the decision of the Ministry of General Education to restrict the distribution of any form of contraceptives within the schools and the expectations that school-going adolescents should only seek SRHR information and uphold abstinence. This expectation was different to what was planned to be taught to adolescents in the RISE youth clubs and be discussed with the parents in the community meetings. It was therefore anticipated that the restriction on distribution of condoms and contraceptives would be an obstacle to encouraging uptake of SRHR interventions. One participant described this as follows:

*“I think we needed to tread very carefully because again you may know the position of the Ministry of Education is that it cannot distribute [condoms and contraceptives]. I don’t think you are allowed to give condoms to the young people in schools, which affects the information that pupils receive … ...So, we needed to be very careful so that we would not create resistance. If we put the issue of contraceptives ahead of us, it would just be an impediment to the whole thing. So that was the fear.” Key Informant interview 8.*

### Socio-cultural norms

At community level, reports from the participants indicated that access and adoption of SRHR information was highly affected by social-cultural norms. Culturally, unmarried adolescents were generally expected to abstain from sexual activities until they are married. Discussing certain SRHR information, such as contraceptives, with unmarried adolescents was also considered unacceptable. Further, SRHR information was affected by religious beliefs and teachings that seemed to support and emphasize societal and cultural norms of promoting abstinence. It was evident from the discussions that participants thought religious and cultural beliefs coupled with limited information would negatively affect uptake of information on contraceptives and sexuality. This could be identified from the description by one participant:

*“Right, it is a very tricky area, like I have said, in that people (are) deeply entrenched in their religious beliefs. So that component is really a big challenge, especially in these rural schools. And to me, what I have seen is that the religious people would rather bury their heads in the sand and assume that it is not happening. We would rather preach abstinence as it is key for them.” Key Informant Interview 11.*

In addition, participants also described the CBHS as consisting of rural communities with low socio-economic status. Most of the participants cited poverty, high numbers of adolescent pregnancies and early marriages and school drop-out as being part of the reasons why these communities were selected to participate in the intervention. Although adolescent pregnancies and early marriages were generally accepted as problems that needed to be addressed, some parents choose to marry off their children in exchange for dowry. In addition, participants also highlighted how adolescents engaging in sexual activities as a way of earning income failed to negotiate for safer sex. This context can be considered as providing enabling conditions that can enhance the acceptability of CBHS interventions that aim to solve these problems, and at the same time hindering acceptability of certain parts of the intervention, such as information related to the use of contraceptives.

### Presence of the CBHS structures

The schools have been working together with the communities and health facilities on certain health-related issues such as immunization programmes. In this collaboration, the role of the school was described by the participants as being a ‘central’ place where health activities are conducted. School-based structures such as the ‘Parents and Teachers Association (PTA)’ were also mentioned as facilitators of the discussion of issues that affect the school and the community. Therefore, even if very little had been done to address the SRHR of adolescents in coordination, it was anticipated that the presence of these existing school-based and community-based structures and previous collaborations could provide an enabling platform where discussions on adolescent SRHR issues could be incorporated.

***“****I think there are other systems that are already there in the school like the parent and teachers committee … we have other systems that are already there in the community, like the Safe Motherhood Action Groups, we have the neighbourhood health committees … .so the approach used in RISE would just be part of that. … So, this component [referring to adolescent SRHR] that we have brought in would contribute to strengthening [the CBHS] by bringing in quite strongly the adolescents” part’ Key Informant Interview 3.*

### Health facility context

The participants described that the health facility context before the implementation of RISE could negatively affect uptake of SRHR interventions. According to the participants, SRH services (including condoms and contraceptives) were offered under the family planning ‘umbrella’ and were easily accessible to married women and men (including married adolescents), but these services were not easily accessible to school-going unmarried adolescents. Two factors were perceived to be affecting accessibility: judgemental perceptions of health workers towards adolescents and lack of confidentiality, as one participant highlighted:

*“A lot of kids will tell you that the only trouble we have is that we have those people responsible for giving out these things (meaning contraceptives) in these communities and clinics. But they really are not confidential. They will secretly go to your parents and say you know what [whispering: your daughter is doing this but don’t tell her I am the one who told you].” Key Informant interview 1.*

This means that although adolescents would be equipped with information on what and where they can seek SRHR services, the prevailing environment at the health facility (before the implementation of RISE) was expected to affect uptake of SRHR services negatively. The combined intervention arm was expected to contribute to transform the CBHS into one that encourages uptake of SRHR among adolescents.

### Presence of economic support for girls

As described above, part of the intervention in the combined intervetion arm offered economic support (namely paying school fees, limited monthly financial support and limited school material) only to the girls who were participating in the intervention and their respective families. Thus, we considered the economic support as being part of the intervention components that created an enabling context in which girls especially those from poorer families were given an opportunity to continue going to school. Our study, however, focused on the components of the intervention that targeted the CBHS and not selected individuals. For example: 1) The youth clubs and community meetings were not meant for only the girls who received financial help but for all school and non-school going boys and girls 2) Community meetings included all community members and not only the parents whose children received financial help form the project. We are also aware that the financial transfer received by the girls participating in the combined intervention arm of RISE could have contributed to making the girls and their families more prone to participate in the activities organized by the RISE team but not per se into the transformation of the CBHS which was our focus. Our interest for this paper, was to focus on the parts of the interventions that targeted the CBHS.

## Outcomes

The two main expected outcomes pointed out by the participants were that interventions such as RISE would lead to: 1) a CBHS where adolescents will have a positive attitude towards SRHR; and 2) a CBHS that encourages uptake of SRHR for adolescents. We considered the two outcomes as domains/features of the overall outcome which was ‘developing an adolescent-responsive CBHS’.

### A CBHS where adolescents show a positive attitude towards SRHR

Participants highlighted that RISE would create spaces (youth clubs and their interactive lessons) that can potentially support adolescents to become confident, increase their self-esteem and make them knowledgeable. This would lead to developing a positive attitude towards SRHR. By a positive attitude, participants meant that adolescents would be able to: abstain from sex while they are still at school; and if sexually active, will be able to seek SRH services that could potentially help them in preventing the negative SRHR outcomes, such as unwanted pregnancies and sexually transmitted infections. Participants also connected these positive attitudes with adolescents’ capability to understand that they were in charge of making decisions related to their SRHR. This included being able to firmly say no to unprotected sex and early marriage. As one participant expressed:

*“We are hoping at individual level that these young people will continue to feel that they are masters of their own destiny, and to know that they have a say on what they would like to happen to themselves and what they don’t want to happen to themselves.” Key Informant Interview 4.*

### A CBHS that encourages uptake of sexual reproductive health service

According to the participants, the intervention was anticipated to create a CBHS where uptake of SRHR services would be easier for adolescents. The participants mentioned different types of SRHR services that they thought adolescents would have access to, and these included SRHR information and linkage to the health facility to access contraceptives if and when they needed them. Adolescents were expected to be able to seek these services because they would have participated in the youth clubs and would be aware and knowledgeable about SRHR issues and how they could deal with them. In the RISE youth club manual this was explicitly stated as:

*“The aims of the youth clubs are to provide them with knowledge about sexual and reproductive health, including modern contraceptives, clarify misconceptions and myths, change beliefs relating to usage of contraceptives; and to empower young people to make good decisions, improve their ability to communicate about sexual and reproductive health with partners, and their ability to negotiate.” Youth Club manual.*

It was anticipated that, the supportive attitudes of parents, teachers, CBHWs and community members (including traditional leaders) towards adolescent SRHR would help to create an enabling environment for adolescents to seek SRHR services at the school, community and health facility levels. Although the health facility staff were generally perceived as being unfriendly to unmarried adolescents seeking SRHR, not everyone agreed. Some participants anticipated that the interventions would contribute to making the local health facility friendlier to adolescents, especially through CBHWs who had participated in the RISE interventions.

## Mechanisms

We identified and grouped mechanisms into 1) mechanisms operationalized at individual level (individual mechanisms) and 2) mechanisms operationalized at group level (collective mechanisms). Although the mechanisms are presented in a sequential way, we consider them nested, and not following a stepwise approach. Awareness and knowledge were expected to reinforce value clarification, which was also expected to lead (or reinforce) individuals into developing a ‘supportive attitude towards adolescent SRHR’. It was also anticipated that these individual mechanisms would in turn reinforce the collective mechanisms, Communication and cohesion; and Social connection and linkages. We present individual and collective mechanisms segregated by actors (highlighted in italics) in which the mechanisms were expected to manifest. The mechanisms are also connected to the interventions, contextual factors and outcomes (Fig. 1).

## Individual level mechanisms

### Awareness and knowledge

*Parents and community members:* Learning and discussing adolescent SRHR issues in the community meetings was expected to increase awareness and knowledge among individual parents. By becoming aware and knowledgeable about adolescent SRHR, participants meant that individual parents and community members would be more conscious of the importance of SRHR issues that affect adolescents in the community. It was also expected that the community meetings would make the parents: aware and knowledgeable about the effects of early pregnancies and early marriages; able to use the acquired SRHR knowledge to talk to their children; and deal with girls who would have already been pregnant or dropped out of school. One participant explained the expected results of the community meetings:

“*… Our assumptions were that surrounding these pregnancies and early marriages were other factors …*. *so, our thoughts were that by providing awareness and education to the community members and parents it would make them know the importance of educating the girl child and taking care of their sexual reproductive needs* ***…***. *that by reaching out at community level we will deal with some of the factors that will be contributing at community level.” Key informant Interview 15.*

*Adolescents:* Increased awareness and knowledge were expected to be triggered by the lessons learnt in the youth clubs. This meant adolescents would be able to know and understand the SRHR issues that affect their lives and what could be done to avoid these SRHR problems, such as early pregnancies, early marriages and school drop-out. With reference to the youth clubs, one participant explained:

“*…*. *but then there could be mental kind of outcomes like self-esteem, confidence − including awareness of SRHR rights; the girls who didn’t know about their rights never thought someone has the right for this, a right to education. Now they see that and the importance of education.” Key informant Interview 9.*

### Value clarification

*Among teachers and CBHWs:* Some participants recounted that interventions like RISE would contribute to developing adolescent responsive CBHS through the trainings offered. At the end of the trainings, teachers and CBHWs were not only expected to have facilitation skills but to also individually clarify their own values towards adolescents’ SRHR. Value clarification was defined by participants as being able to understand and let go of the different societal norms that negatively affected adolescents' SRHR. Value clarification also meant that the teachers and CBHWs would be able to provide adolescents with the much needed SRHR information − and services, if needed − without being constrained by societal and religious beliefs, thus going beyond what is expected at community level (preaching only abstinence to unmarried adolescents) to providing a full range of SRHR information, including issues related to sex, love and contraceptives. Value clarification was also anticipated to trigger teachers and CBHWs into placing adolescent SRHR as one of the important issues that need to be addressed at the school, community and facility levels of the CBHS.

*“So we hope the training on value clarification will make them (referring to the teachers and CBHWs) more understanding … .they will be able to facilitate even the most difficult (SRHR) topics … .so we did spend a lot of time on value clarification so that the teachers and CBHWs understand the importance of teaching the girls and boys all the sexual health lessons, even those they seemed uncomfortable with.” Key Informant Interview 7.*

With their value clarified, the teachers and CBHWs were expected to become effective messengers of SRHR information in the CBHS by sharing information that would arouse parents and community members to reflect on the SRHR issues affecting adolescents. Having their values clarified also meant that the teachers and CBHWs would acknowledge one another and would complement each other during facilitation. Below is how one participant described the results of value clarification:

“*… …*. *the community health worker mentioned earlier on, I think those are very important. So, when they are well qualified and good facilitators, they can really be effective messengers and spread important [SRHR information] and make people reflect on their crisis [referring to adolescent’s pregnancies and early marriages].” Key Informant Interview 6.*

*Among parents and community members:* The discussions facilitated by the teachers and CBHWs during community meetings were expected to trigger value clarification among parents and community members. If parents participated in the community meetings, they would have increased awareness and knowledge on SRHR, and the discussions with others could in turn result in value clarification. Parents whose values were clarified were anticipated to become more willing and open to share SRHR information with their adolescents at household level. One participant explained the expected results from community meetings:

*‘The idea with the community meetings is to initiate discussions that make community members and parents reflect on their practices, hopefully to change norms. Key Informant Interview 6.*

### Supportive attitude

*Parents and community members:* Increased knowledge and awareness and clarifying their own values was assumed to promote a supportive attitude towards adolescent SRHR. At household level, parents were expected to reduce the amount of household work given to the female adolescents, delay marriages and allow their child to continue going to school. A supportive attitude also meant encouraging the sexually active adolescents to seek contraceptives. This type of support was expected from all CBHS stakeholders during and after the intervention. This is captured in the following remarks:

“*…*. *And also, we have been hoping that they [referring to parents and community members] could become more positive to young people accessing contraception and sexual reproductive health services because we know that young people are being sexually active.” Key Informant Interview 6.*

Having a supportive attitude was also expected to mean that each of the individual parents/community members would make a deliberate choice to support interventions that aimed to improve the SRHR of adolescents. As one of the key informants expressed:

*“So, the parents would know why it is important for them to support their daughters to go to school. The idea is that the community becomes an effective partner as we try to support these young people. Otherwise, if you are not careful and don’t involve the community, you could then become their enemy …*. *it is like we are on the same page and speaking the same language.” Key informant interview 15.*

*Teachers and CBHWs:* Teachers and CBHWs were expected not only to support the adolescents by providing information but also provide referrals to appropriate SRHR services required by the adolescents.

## Collective mechanisms

### Communication and cohesion

*Between parents and adolescents:* With their values clarified and a supportive attitude, parents and community members were expected to be more proactive in initiating discussions on SRHR in the CBHS. This would create a platform for adolescents to share their SRHR concerns with their parents. It was expected that conversations around adolescent SRHR would improve communication among all the actors in the CBHS and would continue even after the end of the intervention.

*“Wow …*. *we do think parents will talk more about what affects the girls and discuss with their children on some of the sexual reproductive lessons they learn during community meeting … .so the girls and boys will learn at school and the parents will contribute talking about these issues when they are home.” Key Informant 11.*

*All stakeholders at community level:* Participants also recounted how important it is to create safe shared spaces, such as youth clubs and community meetings, where all stakeholders (community members, teachers and CBHWs and adolescents) could communicate freely about the adolescents’ SRHR. Improved communication would in turn lead to strengthened cohesion between the school, community and health facility, especially on SRHR issues related to adolescents. This is what one of the participants had to say:

*“And then the other thing is that, through the project I think for me I see the enhancement of schools working with communities, with parents (and parents working together). As you know the success of a child in a school can only be realized if families work with the teachers …*. *to create a constant collaboration between parents and the teachers to talk about the welfare of the children.” Key informant Interview 5.*

### Social connections and linkages

This mechanism was expected to be important for adolescents when they wanted to seek information and other SRHR services. By improved social connections, participants explained that adolescents would create stronger and trusting relationships with each other, with the CBHWs and other individuals in the CBHS who would help them if and when they needed to seek SRHR services not provided at school level. According to the participants, CBHWs were anticipated to become a natural link between the school, community and health facility. One key stakeholder explained this:

*“Our thinking was that we needed young people to have a network in case they want to have access to the health service. They need to have someone who they can talk to beyond a teacher …*. *So, a health worker is included to support the young people, and be a major link point to the community health system.” Key informant Interview 12.*

## Discussion

We used the retroductive inferencing logic applied by Mukumbang et al. (2018) to summarize our findings into two alternative ‘extreme’ programme theories (Table 2). Using ‘If …. , Then …. , Because’, we structured the programme theories into testable theories [27]. The first programme theory presents an ideal situation of what would happen if the intervention and actors interacted in an enabling context. The second programme theory presents an alternative theory of what would happen if the conditions in which actors interacted with the interventions were inhibiting. Following the stages of realist evaluation [28, 35], the initial programme theories developed in this phase need to be tested and refined.

**Table 2:** Presentation of the initial programme theories

Programme Theory 1	Programme Theory 2
**IF** interventions that aim to address the SRHR issues of adolescents in the CBHS are supported by existing national policies, socio-cultural norms, existing CBHS structures and functioning local health facilities **THEN** the interventions are likely to make all key stakeholders in the CBHS develop a positive attitude towards adolescents’ SRHR, which will encourage uptake of SRHR services and information **BECAUSE**, at individual level, the intervention will improve awareness and knowledge, clarify the values on SRHR among all the actors (adolescents, CBHWs, teachers and community members), which will at collective level lead to improved communication and cohesion, social connections and linkages among the actors.** AS A RESULT**, the ‘ordinary’ CBHSs will be transformed into systems that are responsive to the SRHR needs of adolescents	**IF** interventions that aim to address the SRHR issues of adolescents in the CBHS are NOT supported by the existing national policies, rejected due to socio-cultural norms and not integrated in the existing CBHS structures and functional local health facilities **THEN** the interventions are likely to make all the key stakeholders develop or maintain a negative attitude towards adolescents’ SRHR, which will discourage uptake of SRHR services and information **BECAUSE** at individual-level the intervention will NOT improve awareness and knowledge, clarify the values on SRHR among all the actors (adolescents, CBHWs, teachers and community members) and at a collective level the intervention will not lead to improved communication and cohesion, social connections and linkages among the actors. **AS A RESULT**, the ‘ordinary’ CBHSs will NOT be transformed into systems that are responsive to the SRHR needs of adolescents

Programme theories were structured using 'If....., Then....., Because.....'

Our study is one of the first studies exploring programme theories of how SRHR interventions implemented in the CBHS can transform the CBHS. These theories are in line with scientific evidence from other contexts that SRHR interventions at the CBHS level must trigger mechanisms at both individual and collective levels for the transformation to occur. Findings reported in a study that used the ecological framework to conceptualize the key elements of ‘approaches that work’ for adolescents’ SRHR, reported that SRH interventions may be more effective if they address SRHR issues at individual, relationship, community and societal levels [23]. According to Svanemyr et al., [23] when all the different levels are targeted, the SRHR programmes contribute to creating an enabling environment for all the actors to adopt the intervention components in a positive manner. Similarly, the stakeholders in our study expected that the intervention would contribute to creating an enabling environment, as it aimed to target all the components of the CBHS.

The identified individual and collective mechanisms anticipated to be triggered among adolescents, parents, community members, CBHWs and teachers suggest that the intervention would go beyond the individual level of providing empowerment, skills and safe spaces (for adolescents) to the relationship and societal levels by creating safe spaces for parents and community members to interact. Further, implementing the intervention in line with existing school and health policies could be seen as a way of strengthening existing policies and laws, thus fulfilling all the requirements of SRHR approaches that work [23]. Our programme theory can therefore be used to inform comparable interventions – which are interventions that aim to achieve similar outcomes [35].

The research team in the RISE trial expected that interventions that target the SRHR of adolescents in the CBHS would significantly be affected by contextual factors, such as the local policies, socio-cultural norms, existing structures in the CBHS and prevailing conditions at the health facility. This was in line with other studies that have highlighted the importance of the context on how adolescents adopt or not SRHR interventions [36, 37]. Depending on contextual conditions and how the actors adopt the intervention, the refined programme theory will probably lie within the broad range of possibilities highlighted by the two ‘extreme’ programme theories. From our literature search, we found no study that has reported on factors or mechanisms that affect the transformation of ‘ordinary’ CBHS (as defined in our study) into systems that are responsive to the SRHR of adolescents, thus making our article distinct. However, we came across studies that have explored factors that affect uptake of SRHR interventions at different arenas of the CBHS (school, community and health facility). Several studies have shown that interventions at community and school level are affected by community norms and beliefs, with school- based SRHR approaches being more effective when supported by the community [21–23, 38, 39]. Evidence shows that prevailing conditions at the health facility, such as the attitude of health workers towards adolescents, affect the uptake of SRH services [21]. Our findings show that the stakeholders anticipate different results from those reported in previous studies due to the intensive value clarification training that was given to CBHWs, who are also key stakeholders at the health facilities in the CBHS.

Although some respondents in our study seemed to suggest a linear relationship between the mechanisms, with one leading into another, the development of the individual and collective mechanisms are more likely to be affected by individual demographics, interpersonal relationships and structural factors, such as political and socio-economic structures [35, 40]. For example, socio-cultural norms have been shown to negatively affect the type of SRHR information given and consequently adoption of SRHR interventions, especially if they include promoting discussions related to sexual matters between parents and unmarried adolescents [36]. In addition, they tend to go beyond promotion of abstinence to promote condom and contraceptive use, which are generally considered culturally a taboo [41, 42]. Although not explicitly stated, the stakeholders seemed to anticipate that the intervention components would, through educating adolescents, help them to challenge the health facility, school and cultural-related barriers that hinder adolescents from seeking SRHR in the CBHS.

In Zambia, like other countries in sub-Saharan Africa, communication in SRHR matters between parents/ community members and adolescents is a challenge [43]. In this study, it was anticipated that engaging parents and community members would potentially improve communication on SRHR-related matters, such as contraceptive use, thus contributing to making the CBHS more responsive towards adolescents; however, this hypothesis needs to be tested. In our programme theory, the stakeholders anticipated that the intervention would be more acceptable when promoting certain SRHR messages − such as abstinence and information − that satisfy the socio-cultural norms than when providing information related to contraceptive use, love, relationships and sex. These anticipations fit with findings from a study conducted in western Ethiopia among adolescents and young people aged 10–24 years, which showed socio-cultural norms as one of the important challenges hindering communication related to SRHR matters. In the Ethiopian study, less than 50% of the participants reported having had discussions with parents about preventive aspects. The percentage reduced further when asked about discussions related to condom use (6.2% males and 3.3% females) and contraceptives (8.2% males and 10% females) [44].

Interventions that target adolescents have often been met with assumptions that adolescents make autonomous decisions regarding their SRHR. This often results in programmes that place the entire SRHR burden on adolescents. We found that the stakeholders recognized the significance of providing SRHR knowledge to all the stakeholders in the CBHS by explicitly engaging the parents, community members, CBHWs, teachers and other stakeholders. Similarly, a Ghanaian study exploring adolescents’ reproductive health knowledge and choices found that adolescent sexual health choices are affected by lack of knowledge, peer pressure and socio-economic factors [21]. In this study, we therefore speculate that interventions like RISE which target all the stakeholders in the CBHS, could potentially be more effective in influencing positive SRHR choices among adolescents than those that place the entire SRHR choice-making burden on adolescents.

The programme theories developed in this article used an SRHR intervention implemented in rural Zambia as a case study [45]. From our findings, we have developed the programme theories, that can be used as a starting point for planning or evaluating other similar interventions. Further, we ensured that the programme theories developed in this study were abstract enough to be tested. According to Pawson [46], when programme theories are abstract in nature, they can be applied to a range of policy areas. One limitation highlighted by Shearn et al. [35], is that programme theories developed using data from stakeholders can become ‘messy’ and unstructured and sometimes not relatable to the different social strata. This limitation was minimized by using the conceptual map to guide the data collection process, which enabled us to solicit for data regarding all the components of the CBHS and the actors that participated in the intervention. Through this process, we ensured that the information collected was structured and related to the different components of the CBHS. Further, the intervention outcomes for RISE were narrower than the outcomes of this realist evaluation, which is the transformation of the CBHS. It is likely that some stakeholders found it challenging to move from the planned RISE outcomes to focusing on the abstract level of how the SRHR interventions could transform the CBHSs. However, we believe this limitation was minimized by explaining the assumptions fully to the participants, that the activities in the combined intervention arm could trigger such a transformation. We tried to minimize social desirability by emphasizing that our evaluation was not an evaluation of RISE but how interventions such as RISE can transform the CBHS. Further, all the interviews were conducted by the first author who was not involved in the implementation and management of the RISE project.

## Conclusion

Although several studies have reported a positive result of interventions that have targeted the different components of the CBHS, there is still a critical gap on how, why and under what circumstance these SRHR interventions can transform the CBHS. Our study is one of the first to document the programme theory of how SRHR interventions can transform ‘ordinary’ CBHSs into systems that are responsive to adolescent SRHR, defined as CBHSs where adolescents will have a positive attitude towards SRHR; and that encourages the uptake of SRHR for adolescents. This article presents two initial testable programme theories. The first theory presumes that adolescent SRHR interventions that are supported by contextual factors are more likely to trigger mechanisms among the different actors that can encourage uptake of the intervention and thus contribute to making the CBHS responsive to the SRHR needs of adolescents. The second and alternative theory suggests that SRHR interventions, if not supported by contextual factors, are less likely to transform the CBHS in which they are implemented. These programme theories will be tested using case studies and the results of the case studies will be reported elsewhere.

## Abbreviations

CBHS: Community-based health system
CBHWs: Community-based health workers
CMOs: Context−Mechanisms−Outcomes
ICAMOs: Intervention−Context−Actors−Mechanism−Outcomes
SRHR: Sexual reproductive health and rights
WHO: World Health Organisation
